# Steroids Regulate CXCL4 in the Human Endometrium During Menstruation to Enable Efficient Endometrial Repair

**DOI:** 10.1210/jc.2016-3604

**Published:** 2017-02-27

**Authors:** Jacqueline A. Maybin, Uma Thiruchelvam, Mayank Madhra, Philippa T.K. Saunders, Hilary O.D. Critchley

**Affiliations:** 1 MRC Centre for Reproductive Health, The University of Edinburgh, Queen’s Medical Research Institute, Edinburgh EH16 4TJ, United Kingdom; 2 MRC Centre for Inflammation Research, The University of Edinburgh, Queen’s Medical Research Institute, Edinburgh EH16 4TJ, United Kingdom

## Abstract

**Context:**

Repair of the endometrial surface at menstruation must be efficient to minimize blood loss and optimize reproductive function. The mechanism and regulation of endometrial repair remain undefined.

**Objective:**

To determine the presence/regulation of CXCL4 in the human endometrium as a putative repair factor at menses.

**Patients/Setting:**

Endometrial tissue was collected throughout the menstrual cycle from healthy women attending the gynecology department. Menstrual blood loss was objectively measured in a subset, and heavy menstrual bleeding (HMB) was defined as >80 mL per cycle. Monocytes were isolated from peripheral blood.

**Design:**

CXCL4 messenger RNA (mRNA) and protein were identified by quantitative reverse transcription polymerase chain reaction and immunohistochemistry. The function/regulation of endometrial CXCL4 was explored by *in vitro* cell culture.

**Results:**

*CXCL4* mRNA concentrations were significantly increased during menstruation. Intense staining for CXCL4 was detected in late secretory and menstrual tissue, localized to stromal, epithelial and endothelial cells. Colocalization identified positive staining in CD68+ macrophages. Treatment of human endometrial stromal and endothelial cells (hESCs and HEECs, respectively) with steroids revealed differential regulation of *CXCL4*. Progesterone withdrawal resulted in significant increases in *CXCL4* mRNA and protein in hESCs, whereas cortisol significantly increased CXCL4 in HEECs. In women with HMB, CXCL4 was reduced in endothelial cells during the menstrual phase compared with women with normal menstrual bleeding. Cortisol-exposed macrophages displayed increased chemotaxis toward CXCL4 compared with macrophages incubated with estrogen or progesterone.

**Conclusions:**

These data implicate CXCL4 in endometrial repair after menses. Reduced cortisol at the time of menses may contribute to delayed endometrial repair and HMB, in part by mechanisms involving aberrant expression of CXCL4.

## Teaser

We studied CXCL4 in endometrial tissue and cells and found that expression was maximal at menses, regulated by cortisol in endothelial cells, and reduced in HMB, consistent with a role in endometrial repair.

The human endometrium displays a remarkable ability to break down and fully repair each month in the absence of pregnancy or lactation. Menstruation is triggered by the withdrawal of the ovarian steroid hormones estrogen and progesterone as the corpus luteum regresses. This results in a local inflammatory response, including leukocyte influx and edema, which culminates in tissue breakdown by matrix metalloproteases and bleeding (1). Much less is known about the mechanisms and regulation of endometrial repair, but the processes involved appear to be similar to those seen with classic wound healing. These involve temporally overlapping phases of inflammation, resolution of inflammation, tissue formation, tissue remodeling, and angiogenesis. In the endometrium, this repair process appears to occur in areas of endometrium adjacent to those where breakdown is in progress (2). Delayed repair of the endometrium at menstruation may cause prolonged heavy menstrual bleeding (HMB), which negatively affects quality of life for many women.

Macrophages have a well-established role in the repair process at multiple tissue sites (3). They engulf foreign or apoptotic material as part of their phagocytic role, and they also secrete many proteases, angiogenic factors, and growth factors (4). Macrophage depletion results in defective repair of skin wounds in the guinea pig (5) and of myocardial injury in mice (6). Endometrial macrophages are present throughout the menstrual cycle but display a substantial increase in number during the perimenstrual phase (7). This increase in the number of tissue resident macrophages is thought to depend on the increase in concentrations of endometrial cytokines that occurs in response to progesterone withdrawal. Cytokines have been implicated in both the recruitment of monocytes into the endometrium and increased proliferation of macrophages *in situ* (7–9). Recent insights into the phenotype of tissue resident macrophages have revealed that both their plasticity and the prevailing tissue microenvironment influence the ability to adopt pro–wound-healing, pro-resolving, and tissue-regenerating phenotypes after injury [reviewed by Wynn and Vannella (10)].

CXCL4 (PF4) is a member of the CXC family that has a role in chemotaxis of neutrophils and monocytes (11, 12). It is unknown whether CXCL4 is an active chemoattractant within human endometrium, but both neutrophils and monocytes are implicated in endometrial repair (13). CXCL4 induces differentiation of peripheral blood monocytes, characterized by prevention of spontaneous apoptosis and promotion of differentiation into macrophages in a tumor necrosis factor-*α* and granulocyte macrophage colony-stimulating factor–independent fashion (14). CXCL4-stimulated differentiation appears to generate a different macrophage phenotype to the classic M1/M2 subtypes (15). Notably, these macrophages lack expression of the scavenger receptor CD163 (15), cannot upregulate heme-oxygenase 1 (15), and do not express the HLA-DR antigen (14) but produce more matrix metalloproteinase (MMP)-7 and MMP-12 protein than other macrophage subtypes (14). In addition, CXCL4 is known to be an angiostatic factor, implicated in inhibition of endothelial cell proliferation (16, 17). CXCL4 has been detected at high concentrations at sites of vascular injury (18) and has downregulated expression of MMP-1 and MMP-3 in human vascular endothelial cells, which may contribute to resolution and repair (19).

Because CXCL4 is thought to have a key role in the regulation of angiogenesis, recruitment of monocytes, and wound healing, we hypothesized that it has a key role in endometrial repair at the time of menstruation (20). Therefore, we conducted a comprehensive analysis of human endometrial biopsy samples and used *in vitro* cell models to examine the regulation of CXCL4 by steroid hormones, including cortisol, because this steroid is thought to play a key role in regulating the local endometrial environment during menstruation. Next, we investigated the effect of CXCL4 on endometrial cells and macrophages. Our results highlight a potential role for this cytokine in the physiologic processes of menstruation and endometrial repair.

## Methods

### Human endometrial tissue collection

Endometrial biopsy specimens (n = 61) were collected with a suction curette (Pipelle; Laboratorie CCD, Paris, France) from women (median age, 42 years; range, 22 to 50 years) attending gynecological outpatient departments across the National Health Service Lothian in Scotland. Participants provided written consent and the Lothian Research Ethics Committee (LREC 07/S1103/29) granted ethical approval. All women reported regular menstrual cycles (21 to 35 days) and no exogenous hormone exposure for 2 months before biopsy. Women with large fibroids (>3 cm) or endometriosis were excluded. Tissue was divided and (1) placed in the RNAlater RNA stabilization solution (Ambion [Europe] Ltd., Warrington, UK), (2) fixed in neutral buffered formalin for wax embedding, and (3) placed in phosphate-buffered saline for *in vitro* culture. Cycle stage was determined by (1) histologic dating [criteria of Noyes *et al.* (21)], (2) reported last menstrual period, and (3) serum progesterone and estradiol concentrations at time of biopsy (Table 1). Samples not consistent for all three criteria were excluded (n = 5).

**Table 1.** **Classification of Endometrial Biopsy Specimens**

Stage of Cycle	Mean Estradiol (pmol/L)	Mean Progesterone (nmol/L)	NMB MBL (Mean; mL)	HMB MBL (Mean; mL)
Proliferative	410 (167–679)	2.8 (1.4–4.6)	N/A	N/A
Early secretory	439 (289–664)	55.4 (26.6–89.9)	N/A	N/A
Mid secretory	585 (301–691)	81.8 (16.1–246.4)	N/A	N/A
Late secretory	275 (59–819)	7.5 (1.1–17.0)	48 (35–62)	200 (85–488)
Menstrual	174 (50–514)	3.4 (1.2–10.6)	40 (26–66)	180 (91–287)

Values in parentheses are the minimum–maximum.

Abbreviation: N/A, not available.

### Objective measurement of menstrual blood loss

A subset of the participants with biopsy specimens collected in the perimenstrual phase agreed to collect their sanitary ware to allow objective quantification of their menstrual blood loss (MBL) (n = 23). Women were provided the same brand of tampon/pad (Tampax^®^/Always^®^; P&G, Weybridge, UK) and verbal and written instructions on collection. Blood loss was measured by using a modified alkaline-hematin method as previously described (22, 23). A measured MBL of >80 mL was classified at HMB and <80 mL as normal menstrual bleeding (NMB). This method was validated in our laboratory by using time-expired whole blood applied to the same sanitary products given to participants.

### Immunohistochemistry for CXCL4

Paraffin sections, 5 μm, were dewaxed and rehydrated. Antigen retrieval was by pressure cooker in sodium citrate (pH, 6) antigen retrieval buffer. Endogenous peroxidase activity was blocked by 3% hydrogen peroxide. Sections were sequentially incubated in avidin and biotin (Vector Laboratories, Burlingame, CA) and protein block (Dako, Cambridge, UK). Rabbit polyclonal CXCL4 antibody (20 µg/mL; ab9561; Abcam, Cambridge, UK) was applied overnight at 4°C. Negative controls were incubated with rabbit IgG (Dako, Cambridge, UK) at the same concentration as the primary antibody. Biotinylated goat anti-rabbit secondary antibody was used at 1:200 (Vector Laboratories, Burlingame, CA). Avidin-biotin-peroxidase complex (ABC-Elite; Vector Laboratories) was applied for 30 minutes, and a liquid diaminobenzidine kit (Zymed Laboratories, San Francisco, CA) was used for detection. The reaction was stopped with distilled water; sections were counterstained with hematoxylin, dehydrated, and mounted with Pertex (Cellpath PLC, Hemel Hempstead, UK).

### Semiquantitative immunoscoring

Localization and intensity of immunostaining were evaluated in late secretory and menstrual endometrial samples from women with objectively measured HMB and NMB by two independent, masked observers. The intensity of staining was graded with a three-point scale (0 = no staining, 1 = mild staining, 2 = strong staining). This was applied to the stromal compartment and endothelial cells. The percentage of tissue in each intensity scale was recorded (24). A value was derived for each cellular compartment by using the sum of these percentages after multiplication by the intensity of staining. Average scores are reported unless a discrepancy of >50 points occurred between observers; in these cases, the tissue was examined together and a consensus score determined.

### Dual immunofluorescence

Endometrial sections were dewaxed, rehydrated, exposed to antigen retrieval, and treated with 3% hydrogen peroxidase as above. For CD68/CXCL4 dual immunofluorescence, normal donkey serum was used as a protein block and the sections were incubated with mouse monoclonal CD68 (macrophage marker) antibody (Dako) at a 1 in 1000 dilution overnight at 4°C. Donkey anti-mouse peroxidase secondary antibody (Abcam) at a 1:750 dilution was applied for 30 minutes, followed by incubation with TSA fluorescein tyramide system (Perkin Elmer, Waltham, MA) for 10 minutes. The sections were incubated with normal donkey serum for 10 minutes, followed by 20 µg/mL rabbit polyclonal CXCL4 antibody (Abcam) overnight at 4°C. Alexa 546 donkey anti-rabbit secondary antibody (Invitrogen, Paisley, UK) was applied at 1:200 for 1 hour, followed by a 4′,6-diamidino-2-phenylindole stain (Sigma-Aldrich, Dorset, UK) for 10 minutes.

CD31/CXCL4 immunofluorescence used Novocastra epitope retrieval solution Ph6 (Novacastra, Leica Biosystems, Newcastle, UK) and the Leica Bond-Max automated immunostainer (Leica Microsystems, Wetzlar, Germany). Normal goat serum was used as a protein block before incubation with CXCL4 antibody (Abcam) at a 1:2000 dilution for 1 hour at 37°C; omission of primary antibody provided negative controls. Goat anti-rabbit secondary antibody (Abcam) was applied before incubation with TSA fluorescein tyramide system for 10 minutes. BOND wash was followed by a BOND epitope retrieval system (Leica Microsystems, Wetzlar, Germany), block with normal goat serum and incubation with mouse monoclonal CD31 (Novacastra, Leica Biosystems) at a 1-in-600 dilution for 1 hour. Goat anti-mouse secondary antibody (Abcam, Cambridge, UK) was applied, followed by a 4′,6-diamidino-2-phenylindole stain (Sigma-Aldrich, Dorset, UK) for 10 minutes. All sections were mounted with Permafluor (Thermo Fisher Scientific, Waltham, MA) and analyzed on a Zeiss LSM710 confocal microscope system (Zeiss, Cambridge, UK).

### Cell culture

Primary human endometrial stromal cells (hESCs) were isolated from midsecretory endometrial tissue (n = 3) by enzymatic digestion as previously described (25). hESCs at passage <6 were plated at a density of 10^6^ cells per well in six-well plates in RPMI medium. Cells were serum starved for 24 hours before treatments. Cells were treated with (1) 10 nM estradiol for 48 hours, (2) 1 µM cortisol for 48 hours, (3) 1 µM progesterone for 6 days, or (4) 1 µM progesterone for 6 days, followed by serum-free media for 48 hours to mimic progesterone withdrawal.

Human endometrial endothelial cells (HEECs) were a gift from Yale School of Medicine (26). Their isolation (27) and phenotype (28) have been previously described. Serum-starved HEECs were treated in an identical manner to hESCs, described earlier.

### Quantitative reverse transcription polymerase chain reaction

Concentrations of messenger RNAs (mRNAs) encoded by *CXCL4* were determined by quantitative reverse transcription polymerase chain reaction (RT-qPCR) (Taqman) analysis. Total RNA from cells and endometrial biopsy samples was extracted by using the RNeasy Mini Kit (Qiagen Ltd, Sussex, UK) according to manufacturer’s instructions: 100-ng RNA samples were reverse transcribed according to standard laboratory protocols (29). A tube with no reverse transcription and a further tube with water were included as controls. RT-qPCR mixtures were prepared containing Taqman buffer (5.5 mM MgCl_2_, 200 μM deoxyadenosine triphosphate; 200 μM deoxycytidine triphosphate; 200 μM deoxyguanosine triphosphate; 400 μM deoxyuridine triphosphate), ribosomal 18S primers/probe (Applied Biosystems, Warrington, UK), and specific forward and reverse primers and probes (CXCL4 forward primer agcctggaggtgatcaagg, reverse primer ccattcttcagcgtggcta, universal probe library number 43; all from Roche Applied Science, Penzberg, Germany) were added for each RT-qPCR reaction. Negative controls (water instead of complementary DNA) were included in each run. RT-qPCR was carried out by using ABI Prism 7900 (Applied Biosystems, Foster City, CA). Forty cycles were completed (3 seconds at 95°C, 30 seconds at 60°C). Samples were analyzed in triplicate by using Sequence Detector, version 2.3 (PE Biosystems, Foster City, CA) with the comparative threshold method. Expression of target mRNA was normalized to RNA loading for each sample using the 18S ribosomal RNA as a reference.

### In cell Western

Following treatments, cells were fixed with 4% NBF for 15 minutes before incubation with blocking buffer (phosphate-buffered saline, normal goat serum, water, and Triton X-100). Cells were treated with rabbit polyclonal anti-CXCL4 (1:25; Abcam) and mouse monoclonal anti–*β*-tubulin (1:1000; Sigma-Aldrich) antibodies overnight at 4°C. Cells were washed before incubation with goat anti-rabbit IRDye 800CW (Molecular Probes, Eugene, OR) and goat anti-mouse Alexa Fluor 680 (LI-COR Biosciences, Lincoln, NE). The LI-COR Odyssey IR Imaging System was used to analyze results.

### Macrophage culture

Peripheral blood was obtained from consenting women (LREC 08/S1103/38) who were taking the combined oral contraceptive pill (n = 9) to avoid natural hormone fluctuations and monocytes extracted, as previously described (9). Monocytes were cultured into RPMI 1640 medium (Sigma-Aldrich) with macrophage colony-stimulating factor (216.21 nM) treatment for 5 days to differentiate the cells into macrophages. Macrophages were then treated with 285.71 nM granulocyte macrophage colony-stimulating factor (to induce an M0 phenotype), 59.17 mM interferon-*γ* (M1 phenotype), 1 μM cortisol (M2 phenotype), estrogen (10 nM), or progesterone (10 nM) for 24 hours. Cells were washed and resuspended in serum-free RPMI medium for 24 hours and centrifuged, supernatant was removed, and cells were frozen for use as conditioned media.

### Chemotaxis assay

Microslides (Ibidi, Martinsried, Germany) were coated with collagen according to the manufacturer’s instructions; collagen was solidified by incubation at 37°C for 30 minutes. The first well of each capillary had peripheral blood monocyte–derived macrophages (PBMCs) in RPMI media (Sigma-Aldrich). The connecting well had 20 ng/mL CXCL4 [Sigma-Aldrich; half maximal effective concentration for CXCL4 as found by Baltus *et al*. in 2005 (11)] in RPMI media; RPMI medium alone was used as a negative control. Movement of PBMCs was measured after 24 hours by using an Axiovert 200 microscope (Zeiss). Distance measured was converted into percentage movement, wherein complete movement would be 100% and no movement, 0%. The experiment was repeated with PBMCs from five different women.

### Statistical analysis

For cell culture, mRNA results are expressed as fold increase, wherein relative expression of mRNA after treatment was divided by the relative expression after vehicle treatment. For tissue data, results were expressed as a quantity relative to a comparator, a sample of placental cDNA. Data are presented as mean ± standard error of the mean and statistically significant differences among raw data (ddCt values) were determined by using Kruskal–Wallis nonparametric test with Dunn multiple comparison posttest. Statistical analysis between women with HMB and NMB at different stages of the cycle was performed by using a two-way analysis of variance with Bonferroni posttest analysis. Student *t* tests were used for immunoscore data. GraphPad Prism software, version 6, was used (San Diego, CA). *P* < 0.05 was considered to represent a statistically significant difference.

## Results

### *CXCL4* mRNA concentrations were increased in menstrual phase human endometrium and CXCL4 localized to epithelial, stromal, and endothelial cells and macrophages

**Figure 1.**
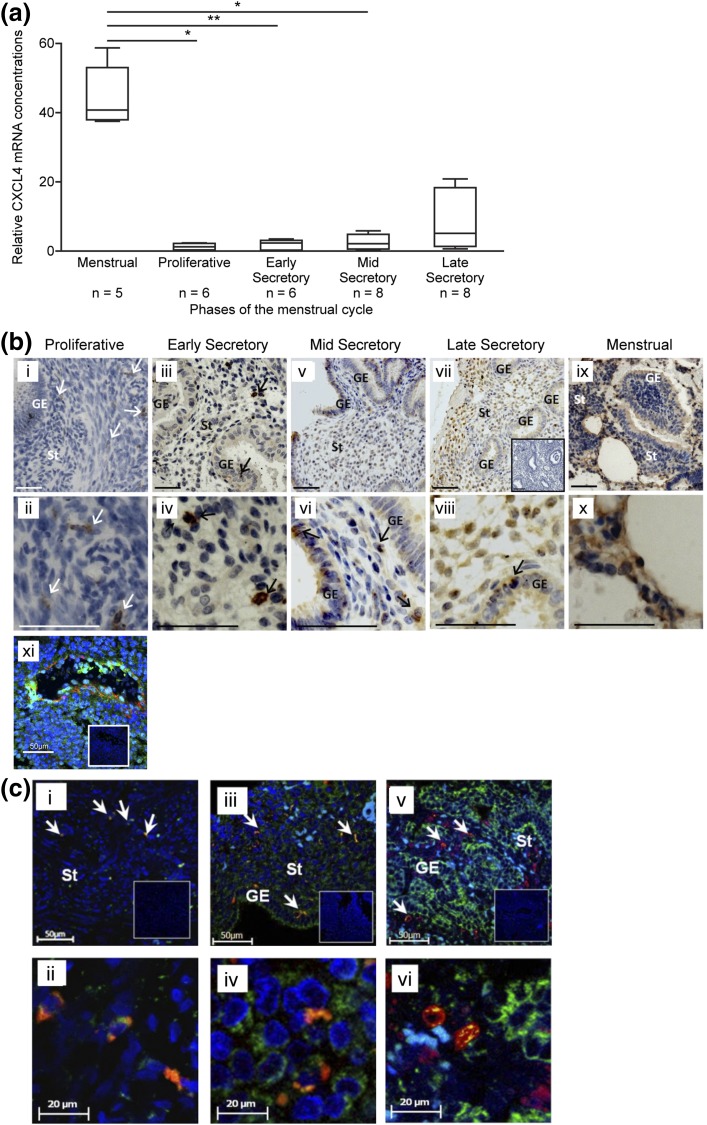
(a) *CXCL4* in whole human endometrial biopsy specimens from across the menstrual cycle reveals maximal expression during the menstrual phase. Each box represents lower quartile, median, and upper quartile. Whiskers display minimum and maximum values. *P < 0.05; **P < 0.01. (b) CXCL4 protein in the human endometrium was localized to the cytoplasm of a few stromal cells during the proliferative phase (i and ii). During the early (iii and iv), mid (v and vi), and late (vii and viii) secretory phases of the menstrual cycle, immunostaining for CXCL4 progressively increased in intensity in both the stroma (St) and secretory glandular epithelium (GE). CXCL4 protein was present throughout the endometrium during the menstrual phase (ix and x) and CXCL4 (green) colocalized to CD31+ endothelial cells (red) in the late secretory phase (xi). Insets depict negative controls. Arrows indicate CXCL4+ cells. Scale bar = 50 µm. (c) CXCL4 (green) colocalizes to CD68+ (red) macrophage cells within the endometrium throughout the menstrual cycle. Proliferative (i and ii), late secretory (iii and iv), and menstrual (v and vi) phases are shown. Insets show negative controls. Arrows indicate colocalized cells.

*CXCL4*-encoded mRNAs were detected in human endometrial tissue biopsy samples throughout the cycle [Fig. 1(a)]. *CXCL4* mRNA concentrations were significantly higher in menstrual biopsy specimens than those from the proliferative (*P* < 0.05), early secretory (*P* < 0.01), and mid-secretory (*P* < 0.05) phases.

Immunohistochemistry detected CXCL4 protein in the cytoplasm of epithelial and stromal cells throughout the menstrual cycle, with an increase in staining intensity noted in endometrium collected from women during the secretory and menstrual phases [Fig. 1(b)]. Dual immunofluorescence revealed positive CXCL4 staining in CD31+ endometrial endothelial cells during the late secretory/menstrual phase [Fig. 1(b, xi)]. We observed intense immunostaining of occasional cells within the stromal compartment throughout the cycle. Dual immunohistochemistry revealed CXCL4 was present in the cytoplasm of CD68+ macrophage cells throughout the menstrual cycle [Fig. 1(c)].

### Endometrial CXCL4 was regulated by progesterone withdrawal and cortisol

**Figure 2.**
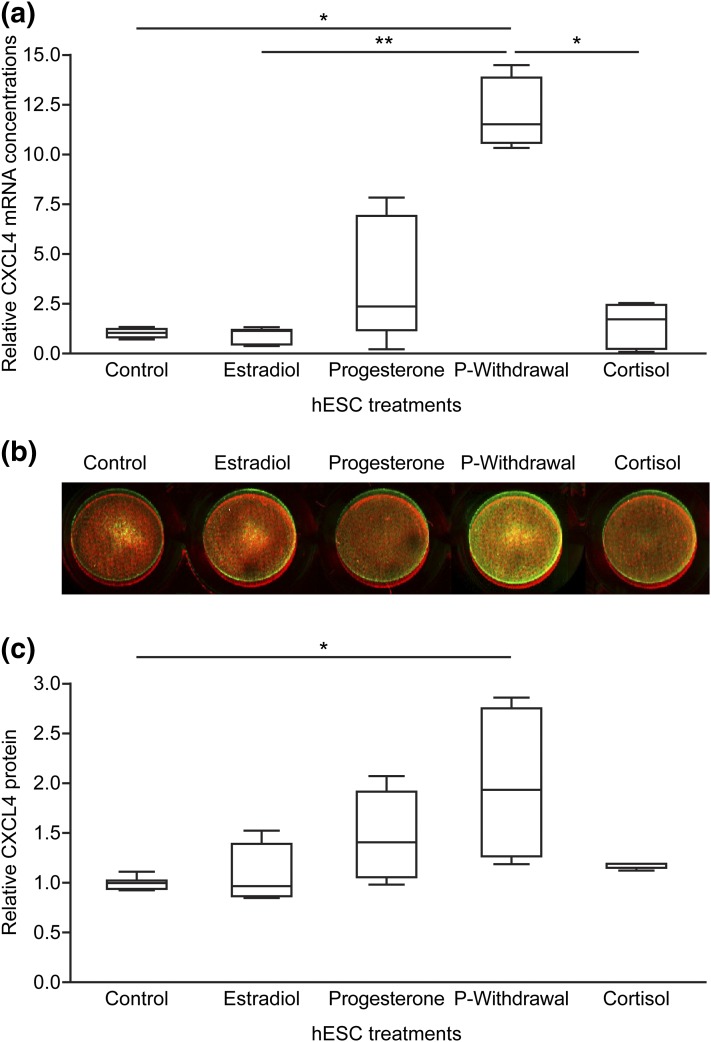
Steroid regulation of CXCL4 in hESCs. Estrogen, progesterone, progesterone withdrawal (P withdrawal), or cortisol treatment of hESCs revealed that progesterone withdrawal significantly upregulated (a) *CXCL4* mRNA concentrations (n = 5) and (b) CXCL4 protein levels detected by in-cell Western blot (n = 4) and quantified by densitometry (c). Green, CXCL4; red, *β*-tubulin. In (a) and (c), each box represents lower quartile, median, and upper quartile. Whiskers display minimum and maximum value. *P < 0.05; **P < 0.01.

After confirming the presence of CXCL4 in endometrial stromal and endothelial cells, we examined its regulation by steroids using primary hESCs and an HEEC cell line. Treatment with 10 nM estradiol mimicked the proliferative phase; 1 µM progesterone, the secretory phase; and sequential progesterone treatment and subsequent removal, the late secretory/menstrual phase. Mounting evidence suggests that cortisol has an important role in the local endometrial environment at menses (9, 20); therefore, additional cells were treated with 1 µM cortisol. hESCs undergoing progesterone-withdrawal treatments showed a significant increase in concentrations of *CXCL4* mRNA compared with those treated with vehicle, estradiol, or cortisol [Fig. 2(a)]. Progesterone withdrawal also significantly increased CXCL4 protein in hESCs compared with vehicle-treated cells [Fig. 2(b) and 2(c)].

**Figure 3.**
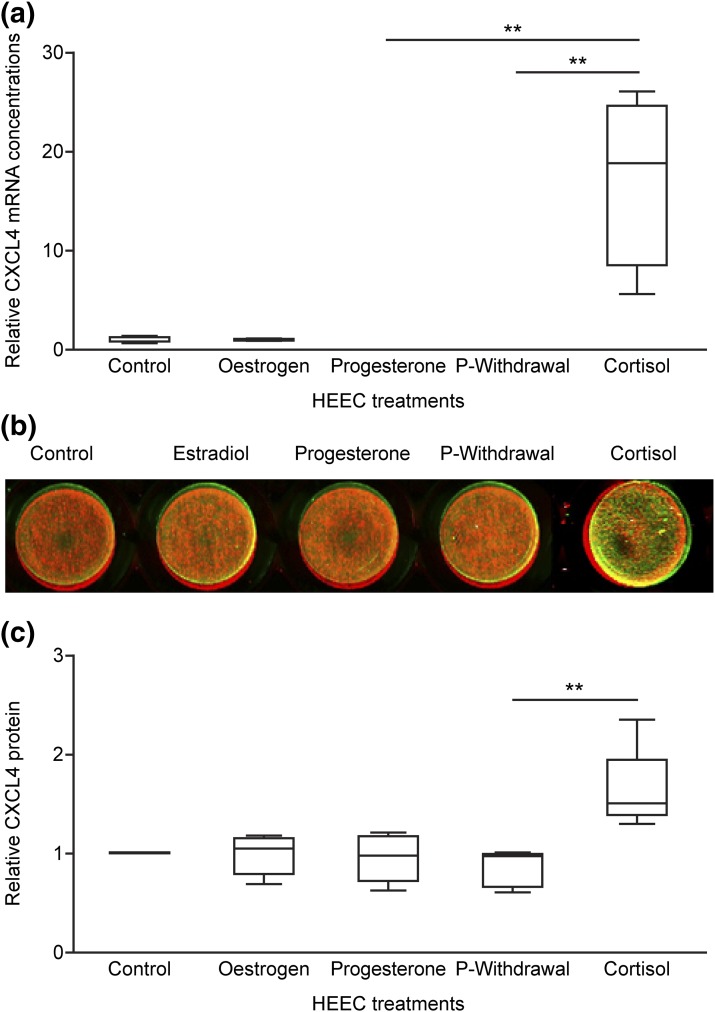
Steroid regulation of CXCL4 in HEECs. Treating HEECs with estrogen, progesterone, progesterone withdrawal (P withdrawal), and cortisol showed that CXCL4 was significantly upregulated by treatment with cortisol at the (a) mRNA level (n = 4) and (b) protein level (n = 4), quantified by densitometry (c). Green, CXCL4; red, *β*-tubulin. In (a) and (c), each box represents lower quartile, median, and upper quartile. Whiskers display minimum and maximum value. **P < 0.01.

Interestingly, CXCL4 regulation in HEECs was different from that detected in hESCs. Cortisol treatment of HEECs displayed maximal increases in concentrations of *CXCL4* mRNA [Fig. 3(a)] and protein [Fig. 3(b) and 3(c)], which were significantly greater than treatment to mimic progesterone withdrawal (*P* < 0.01).

### CXCL4 was significantly decreased in endometrial endothelial cells from women with HMB during menstrual phase

**Figure 4.**
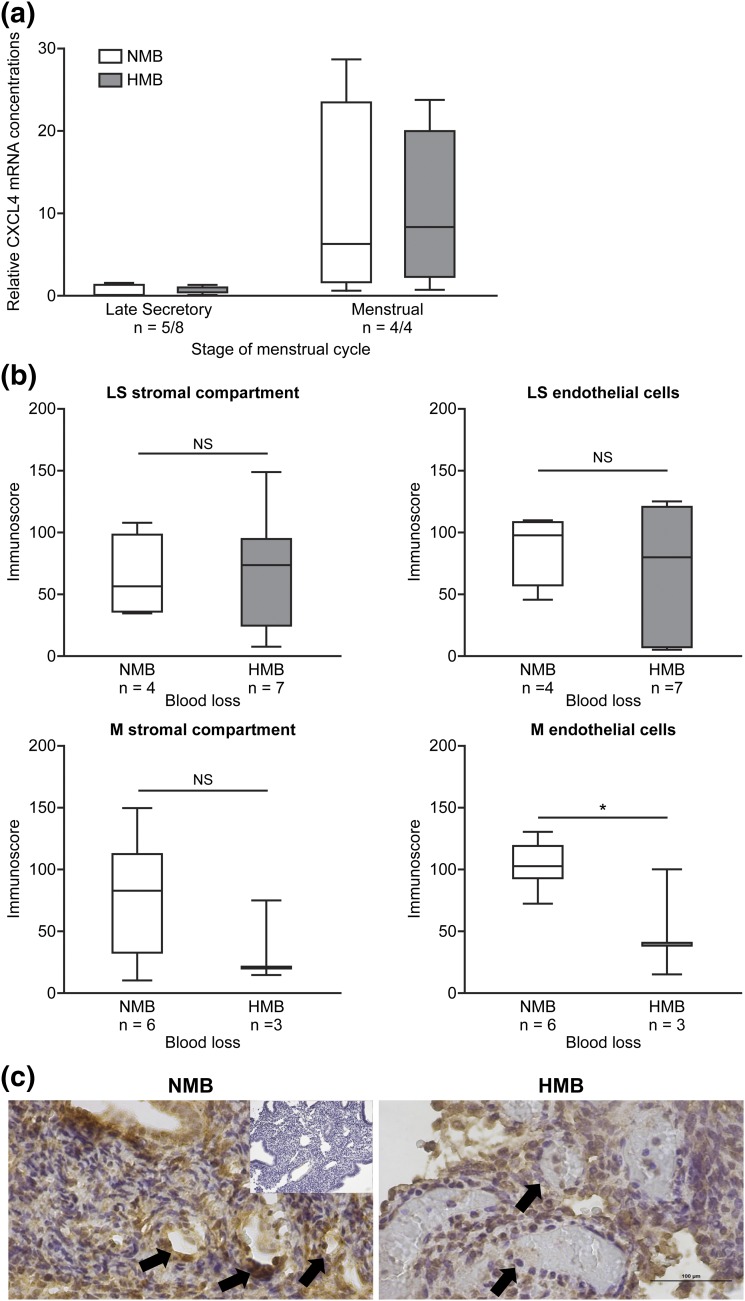
(a) *CXCL4* in late secretory and menstrual endometrial biopsy specimens from women with objectively measured NMB (<80 mL, white bars) and HMB (>80 mL, gray bars). (b) Immunoscoring of CXCL4 staining of the stromal (St) compartment and endothelial cells in late secretory (LS) and menstrual (M) endometrium from women with NMB and HMB. (c) Immunohistochemistry staining of CXCL4 in menstrual endometrium from women with NMB and HMB. Inset shows immunoglobulin G–matched negative control. Arrow, endothelial cells; NS, nonsignificant. In (a) and (b), each box represents lower quartile, median, and upper quartile. Whiskers display minimum and maximum value. *P < 0.05.

Because *CXCL4* mRNA was maximal in endometrium from the late secretory and menstrual phases of the cycle, we compared mRNA concentrations in endometrial tissue homogenates from these two phases, taken from women with objectively measured MBL. Using a blood loss of >80 mL to define HMB, we found no significant differences in mRNA concentrations when comparing women with HMB and NMB [Fig. 4(a)].

Because we determined that regulation of CXCL4 varied in stromal and endothelial cells *in vitro*, we hypothesized that cellular levels of CXCL4 may differ in women with HMB and NMB, despite no significant differences in global endometrial *CXCL4* mRNA concentrations. We examined CXCL4 protein by immunohistochemistry in the endometrium of the late secretory and menstrual phases from women with NMB and HMB. Semiquantitative immunoscoring of the stromal compartment and endothelial cells revealed no significant changes during the late secretory phase between women with NMB and HMB [Fig. 4(b)]. However, menstrual-phase endometrium from women with HMB had significantly decreased CXCL4 staining of endothelial cells compared with tissue from women with NMB (*P* < 0.05) [Fig. 4(b) and 4(c)]. In contrast, menstrual stromal compartment staining was not significantly different in endometrium from women with NMB and those with HMB.

### CXCL4 had augmented chemotactic action on macrophages pre-exposed to cortisol

**Figure 5.**
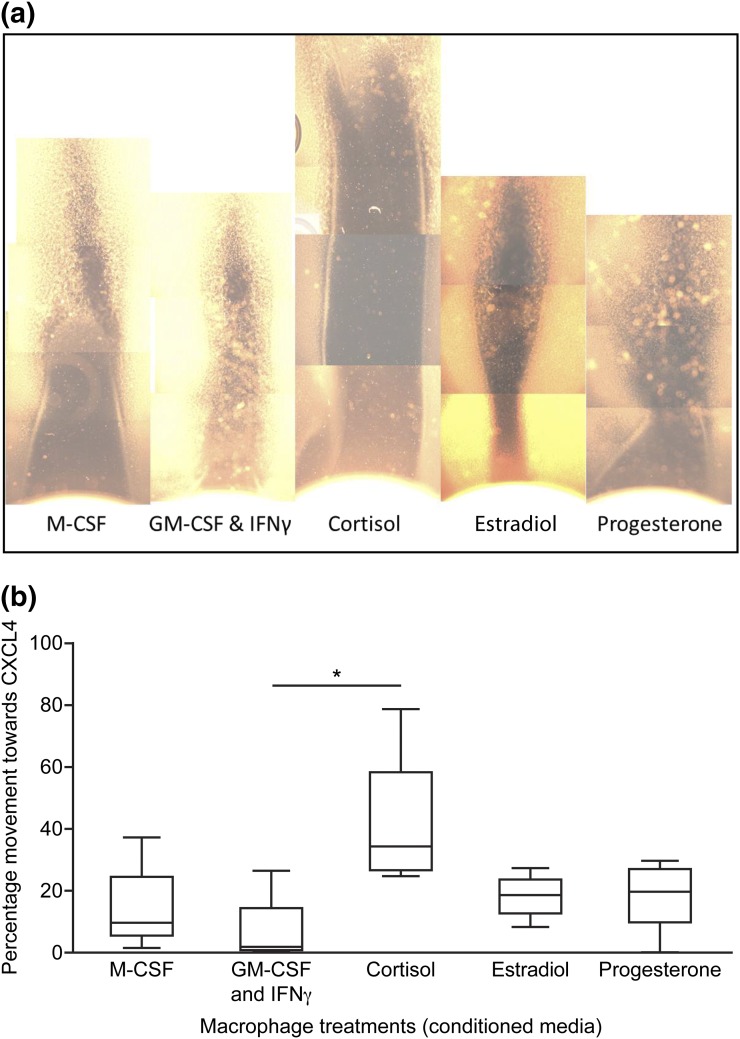
Cortisol-exposed macrophages show increased migration toward CXCL4. (a) Pretreated macrophages [with macrophage colony-stimulating factor (M-CSF) to give an M0 phenotype, granulocyte macrophage colony-stimulating factor (GM-CSF) and interferon (IFN)-*γ* to produce an M1 type, cortisol to give an M2 phenotype, or estradiol or progesterone] were plated opposite CXCL4 and photographed after 24 hours. (b) Measuring distance traveled (as a percentage of total distance) confirmed that macrophages pretreated with cortisol migrated significantly farther than other cells. n = 5 separate patient samples; *P < 0.05.

Because CXCL4 increased at menses and colocalized to macrophage cells, we investigated the effect of CXCL4-induced chemotaxis on different macrophage subtypes. Peripheral macrophages were pretreated to induce different subtypes: M0 (macrophage colony-stimulating factor–pretreated), M1 (granulocyte macrophage colony-stimulating factor– and interferon *γ*–pretreated), and M2 (cortisol-pretreated) macrophages or macrophages exposed to a proliferative phase environment (estradiol-pretreated) or to a secretory phase environment (progesterone-pretreated). These pretreated macrophages were plated into wells opposite CXCL4 on a multichanneled microslide. Cortisol-exposed macrophages migrated toward CXCL4 at a significantly higher rate than any of the other macrophage subtypes [Fig. 5(a) and 5(b)].

## Discussion

This report details the presence of CXCL4 in the human endometrium across the menstrual cycle and reveals that maximal levels are present during menstruation. Steroid regulation of CXCL4 occurs in hESCs, with significant increases after withdrawal of progesterone. In contrast, endometrial endothelial cells do not display an increase in CXCL4 on progesterone withdrawal but do demonstrate significant increases in response to cortisol treatment. Furthermore, we reveal that women with HMB have significantly reduced CXCL4 in endothelial cells in the menstrual phase, consistent with a defective cortisol response at menses (20). Macrophages pretreated with cortisol to induce an M2 phenotype migrate significantly faster toward CXCL4 than M0 and M1 subtypes. These data are consistent with CXCL4 having a key role in endometrial breakdown and repair at menstruation.

CXCL4 is present in the human endometrium during menstruation, with both RT-qPCR and immunohistochemistry being consistent with maximal detection during the menstrual phase. The functional layer of the endometrial breaks down during menses, with repair occurring simultaneously in adjacent areas (2). Therefore, maximal CXCL4 within the endometrium at this time is consistent with involvement in breakdown and repair of the tissue. Expression of the CXCL4 receptor, CXCR3, has been identified as necessary for efficient wound healing (30). Mice lacking CXCR3 had significantly delayed re-epithelialization and delayed repair of the basement membrane following excisional wounds.

Next, we investigated the regulation of CXCL4 in the human endometrium. Because of the dramatic variations observed across the menstrual cycle, we examined steroid regulation of this cytokine. A series of *in vitro* studies revealed that progesterone withdrawal resulted in a significant increase of CXCL4 expression within endometrial stromal cells, consistent with maximal levels during menstruation. HEECs, however, do not express the progesterone receptor (31); hence, it was unsurprising that treatment conditions using progesterone or progesterone withdrawal had no profound effects. However, HEECs are known to express the glucocorticoid receptor (32), and treatment of these cells with cortisol resulted in a significant increase of CXCL4 expression. We have previously shown that local levels of cortisol regulating enzymes increase in human endometrial tissue during menstruation (33). Therefore, two different steroid hormones have the ability to regulate CXCL4 in endometrial cells to increase concentrations of this putative wound repair factor during menstruation.

Because CXCL4 is a putative endometrial repair factor, we examined mRNA concentrations in endometrial tissue sample homogenates from women with HMB and NMB. We hypothesized that women with HMB would have reduced CXCL4 induction during menstruation, leading to inefficient endometrial repair and prolonged, HMB. However, no significant differences in *CXCL4* mRNA concentrations were detected between these two groups of women during the late secretory or menstrual phases.

There are two potential explanations for these findings. First, there may be no deregulation of CXCL4 in women with HMB. However, our results suggested an alternative explanation: that different cell types within the human endometrium have differential regulation of CXCL4 induction, with progesterone withdrawal having a substantial impact on stromal cells and cortisol regulating CXCL4 in endothelial cells. Examination of homogenized whole endometrial biopsy specimens may mask differential expression of CXCL4 within different cell types in women with heavy versus normal menstrual blood loss. Therefore, we examined CXCL4 protein in stromal cells and endothelial cells in women with NMB and HMB during the late secretory and menstrual phases. This revealed that endothelial cell CXCL4 protein was significantly reduced in women with HMB versus NMB during menses, which might be consistent with a defective cortisol microenvironment (33). Our laboratory has previously revealed that the cortisol-inactivating enzyme 11 *β*-hydroxysteroid dehydrogenase-2 is significantly increased in endometrium from women with HMB versus NMB, thereby creating a local glucocorticoid deficiency (20). Therefore, we propose that women with HMB have reduced endometrial cortisol leading to decreased CXCL4 in endothelial cells, which may contribute to increased MBL. CXCL4 is known to have angiogenic properties (18, 19), but its functional role in the endometrium remains to be determined.

CXCL4 is known to be a chemoattractant in many tissues, triggering migration of monocytes and macrophages to sites of inflammation (34, 35). Herein we show that cortisol-treated, M2-like macrophages exhibit increased chemotaxis toward CXCL4 compared with other steroid treated macrophages. This suggests that the microenvironment created by synthesis of CXCL4 may alter immune cell components. It is also notable that cortisol-treated macrophages have been documented to take part in the resolution of inflammation, including removal of apoptotic cells (36). Taken together, these data suggest that CXCL4 may act as a chemoattractant at focal points within the human endometrium that require repair.

In summary, we have identified that CXCL4 is increased in the human endometrium during menstruation, a time consistent with involvement in endometrial repair. Mechanistically, we have revealed that endometrial CXCL4 is regulated by progesterone withdrawal and cortisol. In addition, we reveal that CXCL4 is reduced in endothelial cells of women with HMB at menses. Functionally, CXCL4 appears to have an important role as a macrophage chemoattractant, particularly for macrophages pre-exposed to cortisol. These data implicate CXCL4 as a key player in the physiologic process of endometrial repair after menses.
